# Morphological and molecular evidence support a new species of the genus *Ilocomba* Brescovit, 1997 (Araneae, Anyphaenidae, Anyphaeninae) from the Andes of Colombia

**DOI:** 10.3897/zookeys.1278.162601

**Published:** 2026-04-22

**Authors:** Leonel Martínez, Melisa Eyes-Escalante, Jimmy Cabra-García

**Affiliations:** 1 División Aracnología, Museo Argentino de Ciencias Naturales "Bernardino Rivadavia" CONICET, Avenida Ángel Gallardo 470, CP:1405DJR, C.A.B.A., Buenos Aires, Argentina División Aracnología, Museo Argentino de Ciencias Naturales "Bernardino Rivadavia" CONICET Buenos Aires Argentina; 2 Departamento de Biodiversidad y Biología Experimental, Facultad de Ciencias Exactas y Naturales, Universidad de Buenos Aires, Ciudad Autónoma de Buenos Aires, Argentina Departamento de Biodiversidad y Biología Experimental, Facultad de Ciencias Exactas y Naturales, Universidad de Buenos Aires Ciudad Autónoma de Buenos Aires Argentina; 3 Grupo de Investigación Biodiversidad del Caribe, Departamento de Biología, Universidad del Atlántico, Barranquilla, Colombia Universidad Nacional de Colombia, Sede Bogotá, Facultad de Ciencias, Instituto de Ciencias Naturales Bogotá Colombia; 4 Grupo de Estudios en Sistemática y Conservación, Universidad del Atlántico, Barranquilla, Colombia Grupo de Estudios en Sistemática y Conservación, Universidad del Atlántico Barranquilla Colombia; 5 Universidad Nacional de Colombia, Sede Bogotá, Facultad de Ciencias, Instituto de Ciencias Naturales, Cra 30 45-02, Ciudad Universitaria, Bogotá, 111321, Colombia Grupo de Investigación Biodiversidad del Caribe, Departamento de Biología, Universidad del Atlántico Barranquilla Colombia; 6 Grupo de investigación Biología, Ecología y Evolución de Artrópodos, Departamento de Biología, Universidad del Valle, Cali, Colombia Grupo de investigación Biología, Ecología y Evolución de Artrópodos, Departamento de Biología, Universidad del Valle Cali Colombia

**Keywords:** DNA barcoding, morphology, Neotropics, phylogeny, taxonomy

## Abstract

We describe a new species of the genus *Ilocomba* Brescovit, 1997 from the Andean region of Colombia using an integrative taxonomic approach that combines morphological and molecular data. We assess the phylogenetic position of the new species using four molecular markers: COI, 16S, 28S, and H3. The distinctiveness of the new species, *Ilocomba yotoco***sp. nov**., is supported by its placement in the phylogenetic tree, unique morphological characteristics, and uncorrected pairwise genetic distances. We also provide new morphological data and geographic records for *Ilocomba marta* Brescovit, 1997. Additionally, we present a distribution map of the genus and an updated identification key for all known species of *Ilocomba*.

## Introduction

Colombia ranks among the countries with the highest diversity of genera of Anyphaenidae (Araneae) in the tropical Andes (Dupérré 2023). Of the 59 genera currently recognized within the family, 17 have been documented in the country, representing 28.81% of its total generic diversity (World Spider Catalog 2025). Fourteen of these genera occur within the Andean mountain range, with *Patrera* Simon, 1903 being the most species-rich (Martínez et al. 2021). Notably, the number of anyphaenid species reported from Colombia has increased by over 50% in the recent years (Martínez et al. 2018, 2020, 2021; Oliveira and Brescovit 2021, 2025) Despite these advances, many groups remain understudied and undocumented within Colombian territory.

**Figure 1.**
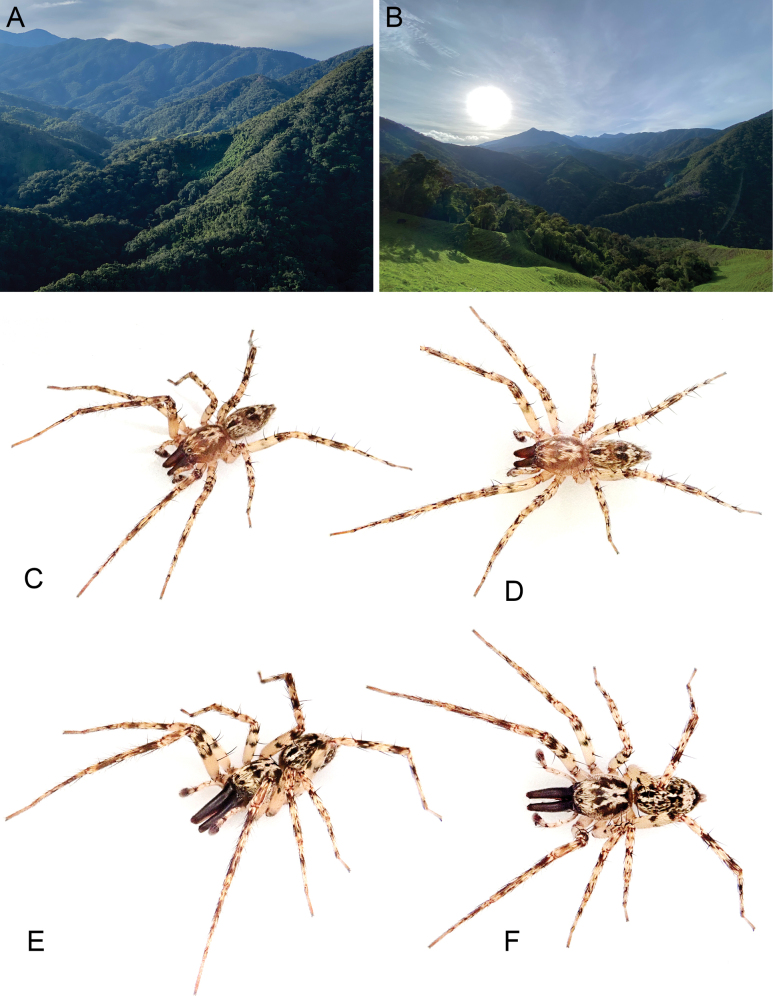
*Ilocomba marta* Brescovit, 1997. A, B. Habitat in the Sierra Nevada de Santa Marta, Colombia; C. Beta male in lateral view; D. Same, in dorsal view; E. Alpha male in lateral view; F. Same, in dorsal view. Images courtesy of Hernan Iuri.

*Ilocomba* is one of the least known genera of Colombian Anyphaenidae (Fig. 1). Brescovit (1997) established the genus with *I. marta* Brescovit, 1997 (based on males and females from the Sierra Nevada de Santa Marta, Magdalena Department) as the type species, and *I. perija* Brescovit, 1997 (based solely on the female holotype from the Serranía del Perijá, Cesar Department) as a second species. According to Brescovit (1997), *Ilocomba* can be recognized by the following combination of characters: a highly reduced retrolateral tibial apophysis, sinuous spermatic ducts, a bifid median apophysis, and a female epigyne with a transverse median septum (Brescovit 1997: 81). Oliveira (2023) recently conducted a phylogenetic analysis of the subfamily Anyphaeninae, which included 34 ingroup genera and 198 morphological characters (76 somatic and 122 genital). In the preferred hypothesis, *Ilocomba* was recovered with moderate support as sister to *Jessica erythrostoma* (Mello-Leitão, 1939).

Here, we obtained Sanger sequences for multiple specimens of *I. marta* from various localities in the Sierra Nevada de Santa Marta, as well as for specimens from Yotoco, Valle del Cauca in southwestern Colombia, which represent a new species. We performed a phylogenetic analysis based on molecular data, combining these new sequences with legacy data from Barone et al. (2024), to assess the phylogenetic placement of the new species. We also provide new distributional and morphological data for *I. marta*, including observations of intrasexual dimorphism, and present the first SEM images for the genus. Finally, we include a taxonomic key and a distribution map for all known species of *Ilocomba*.

## Materials and methods

### Data collections

The material examined is deposited in the Instituto de Ciencias Naturales Universidad Nacional de Colombia, Bogotá (**ICN-Ar**, J. Cabra), Museo Argentino de Ciencias Naturales Bernandino Rivadavia (**MACN-Ar**, M. Ramírez) and Museo de Entomología de la Universidad del Valle, Cali (**MUSENUV**).

### Descriptions and morphology

Specimens from which any type of data was obtained (e.g. images, measurements, tissue samples) are referenced using a voucher code (vchLAM-##), facilitating the association between the generated data and the corresponding specimen. All measurements are provided in millimeters. The descriptive format, including the abdominal spot patterns, follows Brescovit (1997), while the arrangement of ocular patterns and interocular distances follows Petrunkevitch (1925). Coloration was described based on specimens preserved in 80–96% ethanol. Epigynal soft tissues were digested for 24–36 hours in a pancreatin solution following Álvarez-Padilla and Hormiga (2007) and subsequently examined under a microscope while immersed in clove oil.

The anatomical abbreviations used in the text and figures primarily follow Brescovit (1997) and are as follows:

**AB** accessory bulb, SR = seminal receptacles in Brescovit, 1997 and S2 = secondary spermathecae in Oliveira and Brescovit 2021. **ALE** anterior lateral eye.

**AME** anterior median eye

**CD** copulatory duct

**CO** copulatory opening

**CP** cymbial projection

**Cy** cymbium

**d** dorsal

**E** embolus

**FD** fertilization duct

**MA** median apophysis

**MF** median field

**p** prolateral

**PLE** posterior lateral eye

**PME** posterior median eye

**r** retrolateral

**RTA** retrolateral tibial apophysis

**S** spermatheca

**SD** sperm duct

**v** ventral

**alt** altitude (in metres above sea level)

### Digital images and distribution maps

Schematic drawings of female genitalia were produced using a camera lucida mounted on an Olympus BH2 transmitted light microscope. Multifocal images of genitalia, as well as all morphometric measurements, were obtained using a Leica MC-190 HD digital camera attached to a Leica M205A stereomicroscope. For scanning electron microscopy (SEM), dissected structures were sputter-coated with AuPd and imaged under high vacuum using a Zeiss Gemini SEM 360 at the Museo Argentino de Ciencias Naturales (MACN). The figures and plates were edited and prepared in Adobe Photoshop CS v. 12.0. Altitude and coordinates not included in the original labels, were obtained from Google Earth (latitude, longitude, using the WGS84 datum). Data inferred from secondary sources are indicated by [square brackets] in the material examined section. Maps were generated in QGIS (QGIS Development Team 2023) using historical records from Brescovit (1997) and the new records provided here.

### Phylogenetic placement and species boundaries

To investigate the phylogenetic placement of the genus *Ilocomba*, total DNA was extracted from the legs of *I. marta* and the new species described herein. Extractions were performed using either the Wizard Genomic DNA purification kit (Promega), following the manufacturer’s protocol, or a 10% Chelex solution (80 ul per sample), following the protocol outlined by Casquet et al. (2012). The extracted DNA served as a template for the amplification of four molecular makers: the nuclear ribosomal gene 28S rRNA (~700 bp), the nuclear protein-coding gene histone H3 (~300 bp), the mitochondrial cytochrome c oxidase subunit I gene (COI, ~600 bp), and the mitochondrial 16S rRNA gene (16S, ~450 bp), resulting in a total of ~2000 bp of nuclear and mitochondrial DNA. Primers sequences and PCR cycling conditions are provided in Table 1. The PCR products were visualized using agarose gel electrophoresis (1.5–2.0% agarose), purified either with the PCR Clean-Up kit for DNA Sequencing (Biobasic) or enzymatically with ExoSAP, and sequenced bidirectionally by Macrogen Inc. Sequences contigs were assembled using the consed/phred/phrap software suite (Ewing and Green 1998; Ewing et al. 1998; Gordon et al. 1998, 2001) or Sequencher v. 4.1.4. Assembled sequences were compared against the NCBI BLAST database to detect potential contamination. All newly generated consensus sequences were deposited in GenBank. In total, 16 new sequences were produced for this study. The additional sequences were retrieved from GenBank (Table 2).

**Table 1.** List of primers used to amplify DNA sequences of *Ilocomba* species. Abbreviations = AT, annealing temperature.

Marker	Primer name	Primer sequence (5' – 3')	AT (°C)	Reference
COI	L1490 (F)	GGTCAACAAATCATAAAGATATTGG	35 × 48 °C	Folmer et al. (1994)
COI	HCO2198 (R)	TAAACTTCAGGGTGACCAAAAAATCA	35 × 48 °C	Folmer et al. (1994)
16S	LRN13398 (F)	CGCCTGTTTATCAAAAACAT	35 × 47 °C	Simon et al. (1994)
16S	16SAAny (F)	TGTGCTAAGGTAGCATAATCATTTG	35 × 47 °C	Simon et al. (1994)
16S	16SBAny (R)	CCGGTTTGAACTCAGATC	35 × 47 °C	Labarque et al. (2015)
H3	H3aF (F)	ATGGCTCGTACCAAGCAGACVGC	35 × 52 °C	Colgan et al. (1998)
H3	H3aR (R)	ATATCCTTRGGCATRATRGTGAC	35 × 52 °C	Colgan et al. (1998)
28S	28S-O (F)	GAAACTGCTCAAAGGTAAACGG	10 × 52 °C + 25 × 50 °C	Whiting et al. (1997)
28S	28S-C (R)	GGTTCGATTAGTCTTTCGCC	10 × 52 °C + 25 × 50 °C	Whiting et al. (1997)

**Table 2.** Taxon sampling and GenBank accession numbers. Entries in bold were generated in this study.

Species	COI	16S	28S	H3
* Elaver *	KX817506	KX817424	KX817458	KX817557
Anyphaeninae				
*Anyphaena accentuata*	KR559012	KR558880	KR558945	KR558829
*Anyphaena californica*	DQ628605		DQ628660	DQ628633
*Anyphaena pacifica*	KM834979		KM225038	KM225194
*Anyphaenoides clavipes*	KR558955	KR558838	KR558889	KR558772
*Aysha lagenifera*	KY017576	KY015729	KY016917	KY018107
*Aysha proseni*	KR558963	KR558843	KR558897	KR558780
*Buckupiella imperatriz*	KX817504	KX817422	KX817456	KX817555
*Hatitia* sp.	KX817505	KX817423	KX817457	KX817556
*Hibana* sp.	AY297422	AY296713	AY297295	
*Ilocomba marta* LAM 236	PX236108	PX233846		PX378144
*Ilocomba marta* LAM 308	PX236111	PX233844	PX233845	PX388257
*Ilocomba marta* LAM 309	PX236110	PX237211		PX388258
*Ilocomba marta* LAM 416	PX241407			
*Ilocomba marta* LAM 417	PX241363			
*Ilocomba yotoco* sp. nov. LAM 237	PX241572	PX243327		
*Ilocomba yotoco* sp. nov. LAM 418	PX243328			
*Ilocomba yotoco* sp. nov.			PX354550	
*Jessica osoriana*	KR558974	KR558851	KR558908	KR558791
*Otoniela adisi*	KR558984	KR558881	KR558947	
*Otoniela quadrivittata*	MG816011	MG815944	MG815975	
*Xiruana gracilipes*	KR559011	KR558879	KR558944	KR558828
Amaurobioidinae				
*Acanthoceto acupicta*	KR558950	KR558833	KR558884	KR558767
*Sanogasta alticola*	MG815995	MG815927	MG815960	MG815889
*Josa calilegua*	KX817496	KX817414	KX817451	KX817549

The taxon sampling included two representatives of the subfamily Amaurobioidinae, including the genus *Josa*, and nine representatives of Anyphaeninae. The genus *Elaver* (Corinnidae) was defined as root. Phylogenetic relationships were inferred from a concatenated alignment of all sequences (Table 2). Similarity alignments *sensu*Wheeler (2012) were conducted in MAFFT v. 7.299b (Katoh and Standley 2013). The COI and the H3 genes were aligned using the L-INS-i method (command line: mafft—localpair—maxiterate 1000). After alignment, sequences were translated and checked for stop codons using Aliview v. 1.18 (Larsson 2014). The ribosomal genes were aligned using the E-INS-i method (command line: mafft—genafpair—maxiterate 1000), following Wheeler et al. (2017).

Phylogenetic analyses were conducted in IQTREE v. 2.0 (Nguyen et al. 2015). ModelFinder (Kalyaanamoorthy et al. 2017) was used to select the best-fit partition scheme and substitution models (Table 3). Ten independent maximum-likelihood analyses were run, including the calculation of the ultrafast Bootstrap (UFBoot) (Hoang et al. 2018) and the Shimodaira–Hasegawa approximate likelihood-ratio test (SH aLRT) (Guindon et al. 2010), using the following command: iqtree2 -s concat.nex -spp partition.nex.best_scheme.nex -bb 1000 -alrt 1000 -pers 0.2 -nstop 1000. In addition, a maximum parsimony analysis was conducted using TNT v. 1.6 (Goloboff and Morales 2023). All character transformations were equally weighted, and gaps were treated as missing data. Tree searches employed new technology search algorithms using the following TNT command: xmu: hit 50 rep 5 ratchet 50 fuse 20 drift 20. Bootstrap resampling frequencies were estimated with: xmu: rep 5 ratchet 50 fuse 20 drift 20; resample boot rep 1000 freq from 0 [xmult].

**Table 3.** Partitioning scheme and nucleotide substitution models chosen in ModelFinder for phylogenetic analyses.

Subset	Partition names	Model
1	16S	GTR+F+G4
2	28S, H3 codon 1, H3 codon 2, H3 codon 3	TIM2+F+R3
3	COI codon 1, COI codon 2	K3Pu+F+I+G4
4	COI codon 3	GTR+F+I+G4

*Ilocomba yotoco* sp. nov. was delimited based on (1) detailed morphological comparison with previously described *Ilocomba* species, (2) phylogenetic position, and (3) uncorrected pairwise genetic distances (COI). The latter were calculated using ape 5.0 (Paradis and Schliep 2019).

## Results

### Phylogenetic placement and species boundaries

**Figure 2.**
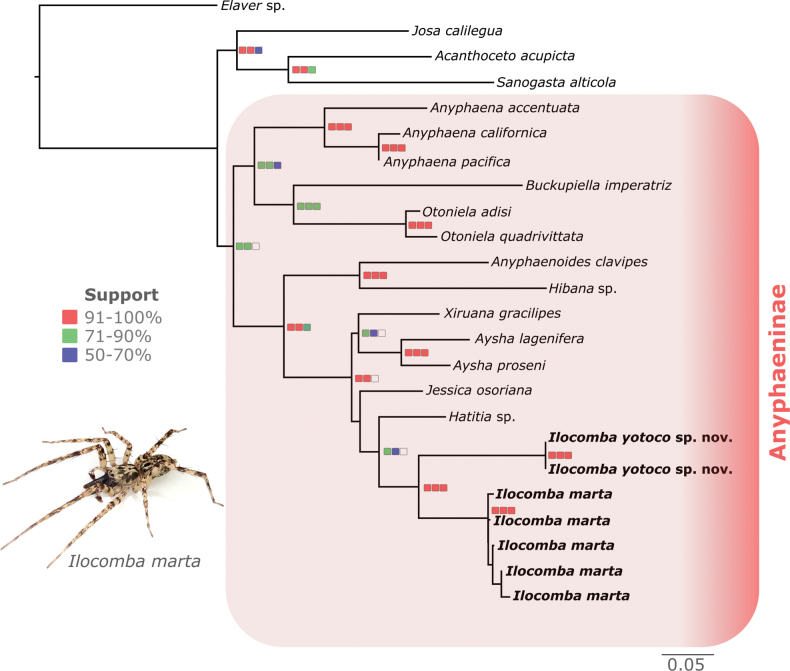
Phylogenetic relationships among selected Anyphaeninae genera and position of *Ilocomba* based on a concatenated dataset (28S, H3, COI, 16S) inferred using maximum likelihood. Specimen image: *Ilocomba marta* male from Sierra Nevada de Santa Marta. Colored squares at nodes indicate branch support values for SH-aLRT (left), ultrafast bootstrap (center), and parsimony bootstrap (right): red = 91–100%, green = 71–90%, blue = 50–70%. The new species *Ilocomba yotoco* sp. nov. and conspecific sequences of *Ilocomba marta* Brescovit, 1997 are highlighted.

*Ilocomba yotoco* sp. nov. was recovered as sister to *I. marta* with high support in both maximum likelihood (ML) and maximum-parsimony (MP) analyses (Fig. 2). In the ML analysis, *Hatitia* was strongly supported as sister to *Ilocomba*. In the MP analysis, however, equally parsimonious topologies placed *Ilocomba* either as sister to (*Hatitia*, *Xiruana*) or to (*Hatitia* (*Jessica*, *Xiruana*)).

The interspecific COI distance among *Ilocomba* species ranged from 6.7% to 8.3%, whereas the intraspecific distance ranged from 0% to 3.1% (Table 4). The maximum interspecific distance among species of Anyphaeninae was 17.8%, observed between *Buckupiella imperatriz* (Brescovit 1997) and *Sanogasta alticola*. Excluding the *Ilocomba* species, the minimum interspecific distance among Anyphaeninae was 7.4%, between *Jessica osoriana* (Mello-Leitão 1922) and *Xiruana gracilipes* (Keyserling 1891) (Table 4).

**Table 4.** Percent uncorrected pairwise distances between COI sequences of Anyphaeninae species. Species codes: 1: *Acanthoceto acupicta*, 2: *Anyphaena accentuata*, 3: *Anyphaena californica*, 4: *Anyphaena pacifica*, 5: *Anyphaenoides clavipes*, 6: *Aysha lagenifera*, 7: *Aysha proseni*, 8: *Buckupiella imperatriz*, 9: *Hatitia* sp., 10: *Hibana* sp., 11-15: *Ilocomba marta*, 16-17: *Ilocomba yotoco* sp. nov., 18: *Jessica osoriana*, 19: *Josa calilegua*, 20: *Otoniela adisi*, 21: *Otoniela quadrivittata*, 22: *Sanogasta alticola*, 23: *Xiruana gracilipes*. Abbreviations: GBA: GenBank accession number.

Species & GBA	1	2	3	4	5	6	7	8	9	10	11	12	13	14	15	16	17	18	19	20	21	22
1 (KR558950)	---																					
2 (KR559012)	0.13	---																				
3 (DQ628605)	0.15	0.09	---																			
4 (KM834979)	0.14	0.09	0.01	---																		
5 (KR558955)	0.14	0.13	0.12	0.12	---																	
6 (KY017576)	0.14	0.13	0.14	0.13	0.14	---																
7 (KR558963)	0.13	0.12	0.12	0.12	0.12	0.08	---															
8 (KX817504)	0.14	0.14	0.16	0.16	0.16	0.17	0.15	---														
9 (KX817505)	0.13	0.13	0.14	0.13	0.12	0.12	0.09	0.16	---													
10 (AY297422)	0.14	0.12	0.13	0.13	0.12	0.13	0.11	0.14	0.12	---												
(11) PX236111	0.12	0.10	0.13	0.13	0.12	0.10	0.09	0.14	0.09	0.12	---											
(12) PX236110	0.12	0.10	0.13	0.13	0.12	0.10	0.09	0.13	0.09	0.11	0.00	---										
(13) PX236108	0.12	0.11	0.14	0.13	0.13	0.11	0.09	0.14	0.10	0.12	0.01	0.02	---									
(14) PX241407	0.12	0.10	0.14	0.13	0.12	0.11	0.09	0.14	0.10	0.12	0.01	0.01	0.01	---								
(15) PX241363	0.12	0.11	0.13	0.13	0.12	0.10	0.10	0.15	0.10	0.12	0.02	0.02	0.03	0.03	---							
(16) PX241572	0.13	0.12	0.12	0.12	0.13	0.10	0.09	0.15	0.10	0.11	0.08	0.07	0.08	0.08	0.07	---						
(17) PX243328	0.13	0.12	0.12	0.12	0.13	0.10	0.09	0.15	0.10	0.11	0.08	0.07	0.08	0.08	0.07	0.00	---					
18 (KR558974)	0.12	0.12	0.13	0.12	0.12	0.09	0.08	0.14	0.09	0.12	0.09	0.09	0.10	0.09	0.10	0.09	0.09	---				
19 (KX817496)	0.13	0.11	0.12	0.12	0.12	0.12	0.10	0.14	0.11	0.10	0.12	0.12	0.12	0.12	0.12	0.12	0.12	0.12	---			
20 (KR558984)	0.13	0.09	0.11	0.10	0.12	0.12	0.09	0.13	0.12	0.11	0.11	0.11	0.11	0.11	0.12	0.10	0.10	0.11	0.11	---		
21 (MG816011)	0.13	0.11	0.10	0.10	0.11	0.12	0.10	0.13	0.13	0.12	0.12	0.11	0.12	0.12	0.12	0.10	0.10	0.11	0.12	0.03	---	
22 (MG815995)	0.15	0.14	0.15	0.14	0.14	0.15	0.14	0.18	0.16	0.15	0.17	0.16	0.17	0.17	0.17	0.16	0.16	0.14	0.15	0.15	0.14	---
23 (KR55901)	0.13	0.12	0.14	0.14	0.12	0.10	0.09	0.14	0.09	0.12	0.09	0.09	0.10	0.10	0.09	0.10	0.10	0.07	0.11	0.12	0.13	0.15

### Taxonomy

**Family Anyphaenidae Bertkau, 1878**

**Subfamily Anyphaeninae Bertkau, 1878**

#### Genus Ilocomba

Taxon classificationAnimaliaAraneaeAnyphaenidae

Brescovit, 1997

C868A78F-D570-5766-96DE-45CA130ADD05

Ilocomba Brescovit, 1997: 82, type species (by original designation): I. marta Brescovit, 1997: 82, figs 204–209.

##### Diagnosis.

Males of *Ilocomba* Brescovit, 1997 can be recognized by the following combination of characters: a well-developed cymbial projection at the base of the cymbium (Figs 5B, 5C, 6A, 6B, 14A, 14B); a rigid embolus, inserted prolatero-medially on the tegulum and directed straight (Figs 5A, 7A, 12A, 13A, 15A, 15B); a bifid median apophysis divided into two distinct branches: a dorsal branch that is elongate, thin, and distally hook-shaped, and a ventral branch that is robust and more heavily sclerotized (Figs 5A, 7A–E, 12A, 13A, 15A–E); and a markedly reduced retrolateral tibial apophysis (Figs 6A–C, 14B, 14C). Females are distinguished by having large spermathecae with very short copulatory ducts, approximately half the length of the spermathecae, inserted anteriorly, and an epigyne bearing posterior lateral sclerotizations (Figs 5F, 5G, 8A–D, 12F, 12G, 13C, 13D).

##### Composition.

Three species, *Ilocomba marta* Brescovit, 1997, *Ilocomba perija* Brescovit, 1997 and *Ilocomba yotoco* Martínez and Cabra-García sp. nov.

##### Intrasexual dimorphism in *Ilocomba*.

**Figure 3.**
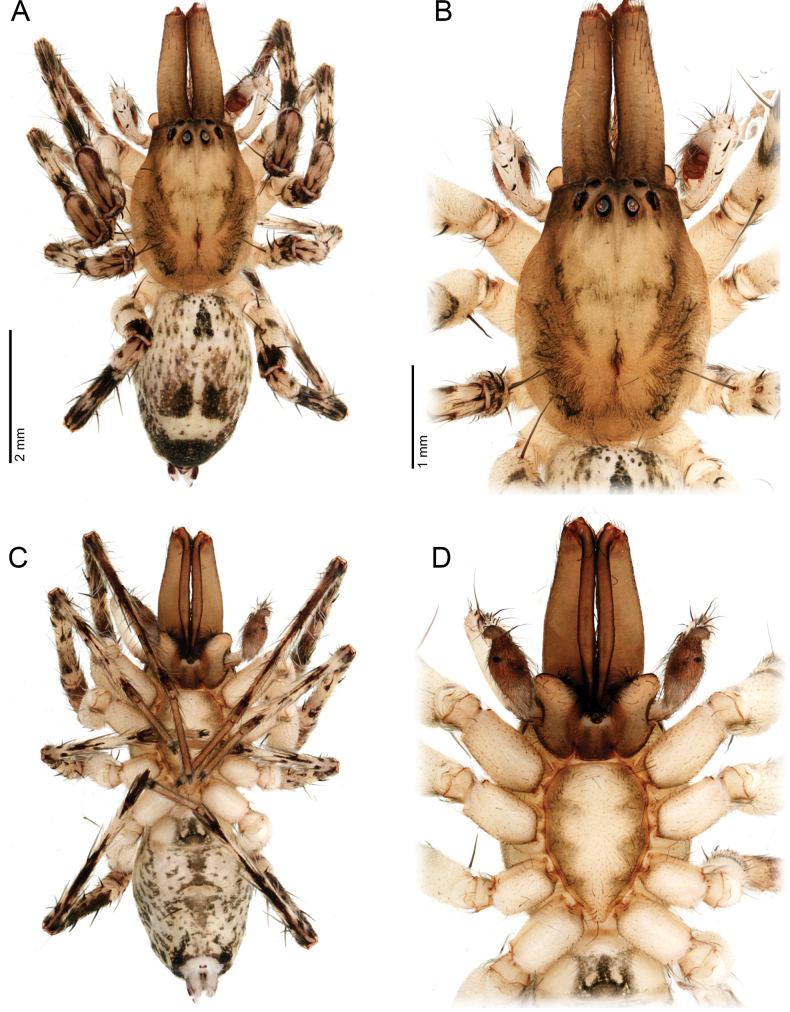
*Ilocomba marta* Brescovit, 1997, alpha male (ICN-Ar 13816; vchLAM-308). A. Habitus, dorsal view; B. Carapace, dorsal view; C. Habitus, ventral view; D. Sternum.

Extensive sampling of *Ilocomba marta*, along with a representative series of *I. yotoco*, revealed clear intrasexual variation among males of both species (Figs 1C, 1D, 17). Two distinct male morphotypes, alpha and beta, were identified, differing primarily in size, length of the chelicerae, and orientation. In alpha males, the chelicerae are markedly elongated; the fang is significantly longer than in beta males, reaching nearly twice its length, while the paturon may be up to three times longer than that of beta males (Figs 3A–D, 10A–D).

**Figure 4.**
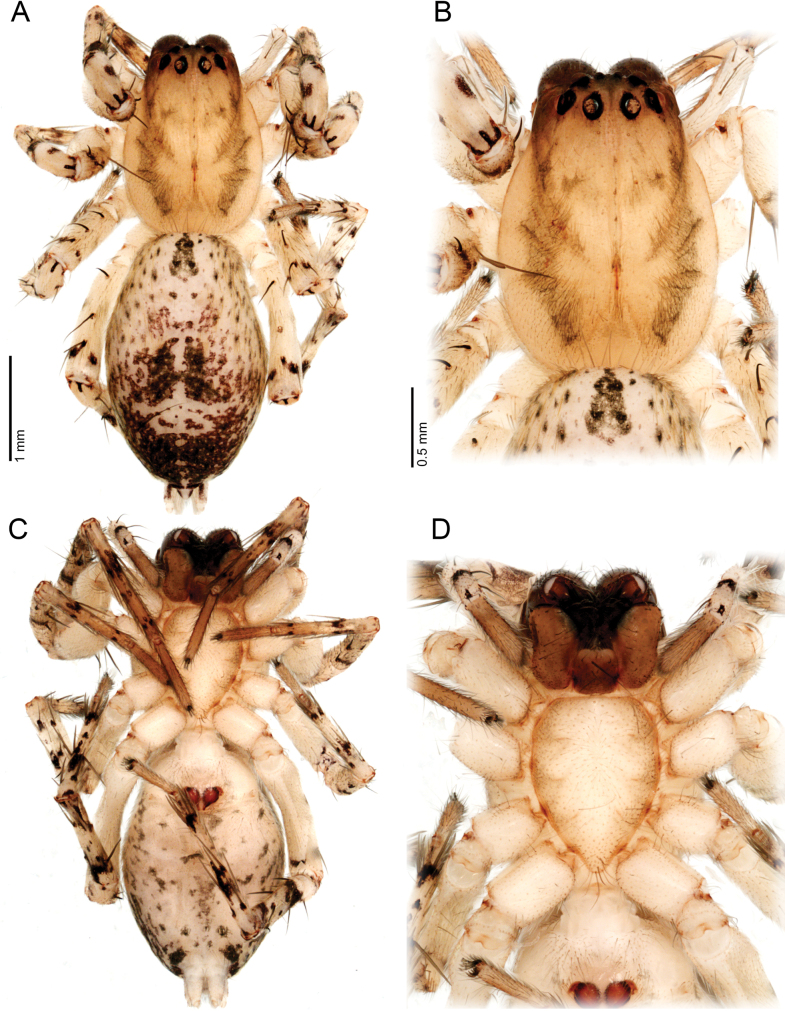
*Ilocomba marta* Brescovit, 1997, female (ICN-Ar 13817; vchLAM-309). A. Habitus, dorsal view; B. Carapace, dorsal view; C. Habitus, ventral view; D. Sternum.

In contrast, beta males possess small, vertically oriented chelicerae, closely resembling those of females; the fang is nearly equal in length to the paturon (Figs 4A–D, 9A–D, 11A–D, 16A–D). The anterior legs of alpha males are slightly longer and more densely setose. Some variation in coloration was observed, particularly among populations of *I. marta*, but no consistent patterns were associated with the male morphotypes. Despite these pronounced somatic differences, no variation in genital morphology was detected between alpha and beta males in either species.

### Key to species of *Ilocomba*

**Table:** 

1	Males (that of *I. perija* unknown)	**2**
–	Females	**3**
2	CP sub-squared, granulate; ventral branch of MA subtriangular; RTA rudimentary	***Ilocomba marta* Brescovit, 1997** (Figs 6A, 6B, 7A–D)
–	CP tubular, horn-shaped, smooth; ventral branch of MA strongly sclerotized, scale-shaped; RTA short but not reduced	***Ilocomba yotoco* sp. nov**. (Figs 14A, 14B, 15A–D)
3	CD almost as long as S; S subrounded, anteriorly elongated	***Ilocomba yotoco* sp. nov**. (Figs 12F, 12G, 13C, 13D)
–	CD shorter than S; S rounded, not elongated anteriorly	**4**
4	CD wide; anterior depression wider than long	***Ilocomba perija* Brescovit, 1997** (see Brescovit 1997: 84, figs 210–211)
–	CD thin; anterior depression almost as long as wide	***Ilocomba marta* Brescovit, 1997** (Figs 5F, 5G, 8A)

#### Ilocomba marta

Taxon classificationAnimaliaAraneaeAnyphaenidae

Brescovit, 1997

4DF3F6FC-D0B4-530B-A5D5-8ABC3B1D7EAC

Figs 1C–E, 3–9, 17A–C

Ilocomba marta Brescovit, 1997: 82, figs 204–209 (male holotype and paratypes from San Sebastian de Rabago [10°52'00"N, 73°43'00"W], Sierra Nevada de Santa Marta, Magdalena Colombia, 1–10.V.1968, B. Malkin leg., deposited in AMNH, examined).

##### Other material examined.

**Colombia**. 2 ♂♂, 3 ♀♀; Cesar, Sierra Nevada de Santa Marta, Paso Bella Vista; [10°50'16.0"N, 73°50'09.0"W]; alt. 3760 m; 7 Dec. 1978; H. Sturm leg.; MCZ-IZ 171000 • 1 ♂; Magdalena, Ciénaga, Sierra Nevada de Santa Marta, San Pedro, Hacienda Hierba Buena (Casa de Don Pablo), bordeando el potrero adyacente a la bajada de la casa; alt. 2177 m; 11 Jun. 2024; Conv. 890 ICETEX team leg.; secondary cloud forest, beating; ICN-Ar 13816 vchLAM-308 • 1 ♀; same data as for preceding; ICN-Ar 13817 vchLAM-309 • 1 ♂; same data as for preceding; ICN-Ar 13818 vchLAM-416 • 1 ♀; same data as for preceding; ICN-Ar 13819 vchLAM-417 • 3 ♂♂, 1 ♀; same data as for preceding; ICN-Ar 13820 • 2 ♂♂; same data as for preceding; ICN-Ar 13821• 1 ♂; Magdalena, Santa Marta, Minca, Sierra Nevada de Santa Marta, Onaca, Camino cerca al Hostal Luisito; (11°6'31.53"N, 74°3'37.73"W); alt. 2388 m; 15 Aug. 2024; Conv. 890 ICETEX team leg.; low secondary vegetation, beating; ICN-Ar 13822 vchLAM-431 • 1 ♂; same data as for preceding; ICN-Ar 13823 vchLAM-432 • 1 ♀; same data as for preceding; ICN-Ar 13824 vchLAM-433 • 2 ♂♂, 6 ♀♀, 5 imm.; same data as for preceding; ICN-Ar 13825 • 1 ♂, 1 ♀; same data as for preceding; ICN-Ar 13826 • 1 ♀; same data as for preceding; ICN-Ar 13827 • 1 ♀; same data as for preceding; ICN-Ar 13828 • 7 ♂♂, 3 ♀♀; same data as for preceding; ICN-Ar 13829.

##### Diagnosis.

**Figure 5.**
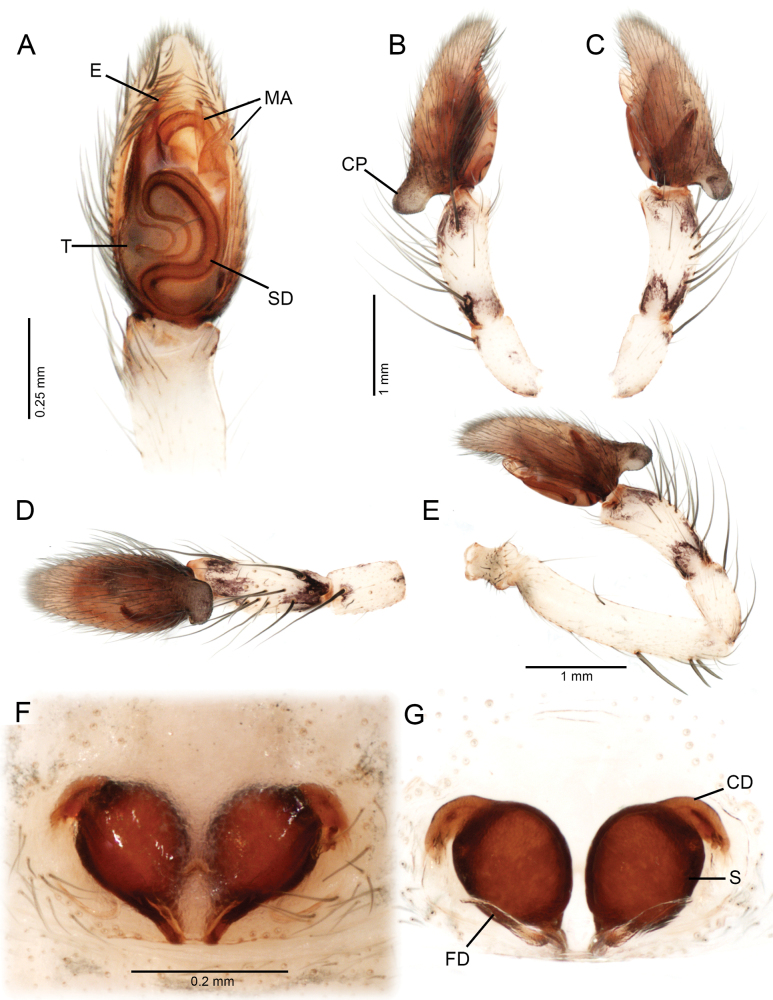
*Ilocomba marta* Brescovit, 1997, male (ICN-Ar 13816; vchLAM-308), left palp. A. Ventral view; B. Prolateral view; C. Retrolateral view; D. Dorsal view; E. Retrolateral view, whole palp. Female (ICN-Ar 13817; vchLAM-309), genitalia; F. Ventral view; G. Dorsal view.

**Figure 6.**
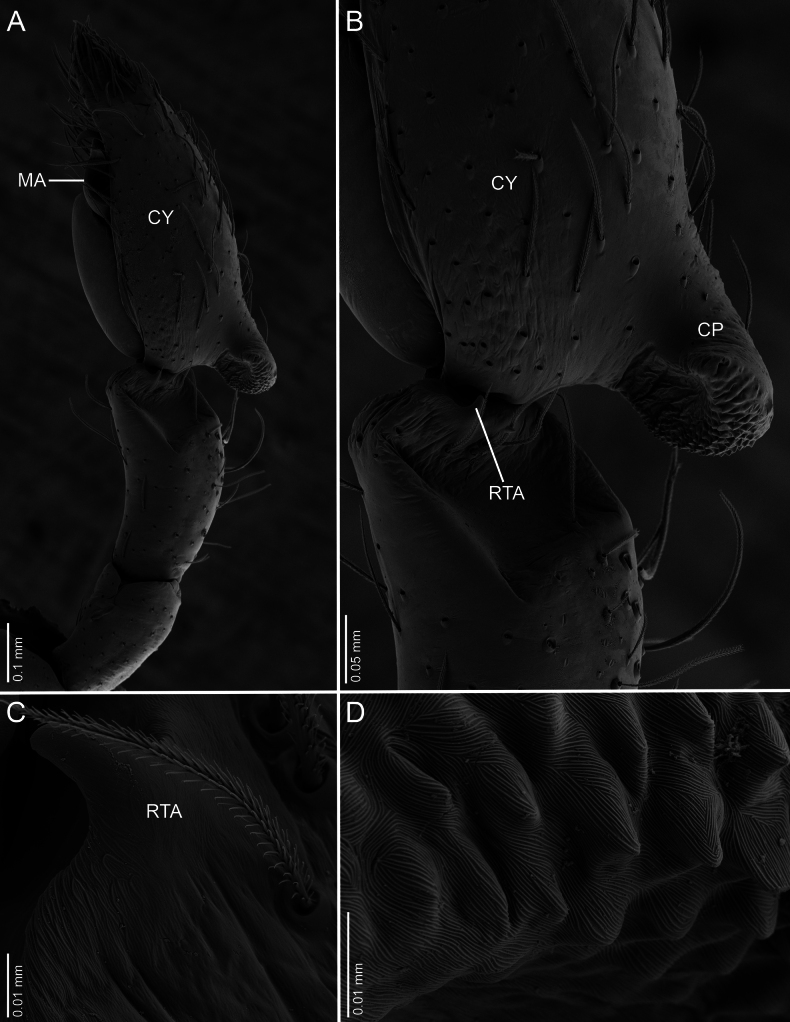
*Ilocomba marta* Brescovit, 1997, male (ICN-Ar 13819), left palp, SEM images. A. Retrolateral view; B. Same, closed-up, details of the cymbial projection; C. Retrolateral tibial apophysis; D. Cuticular detail of cymbial projection.

**Figure 7.**
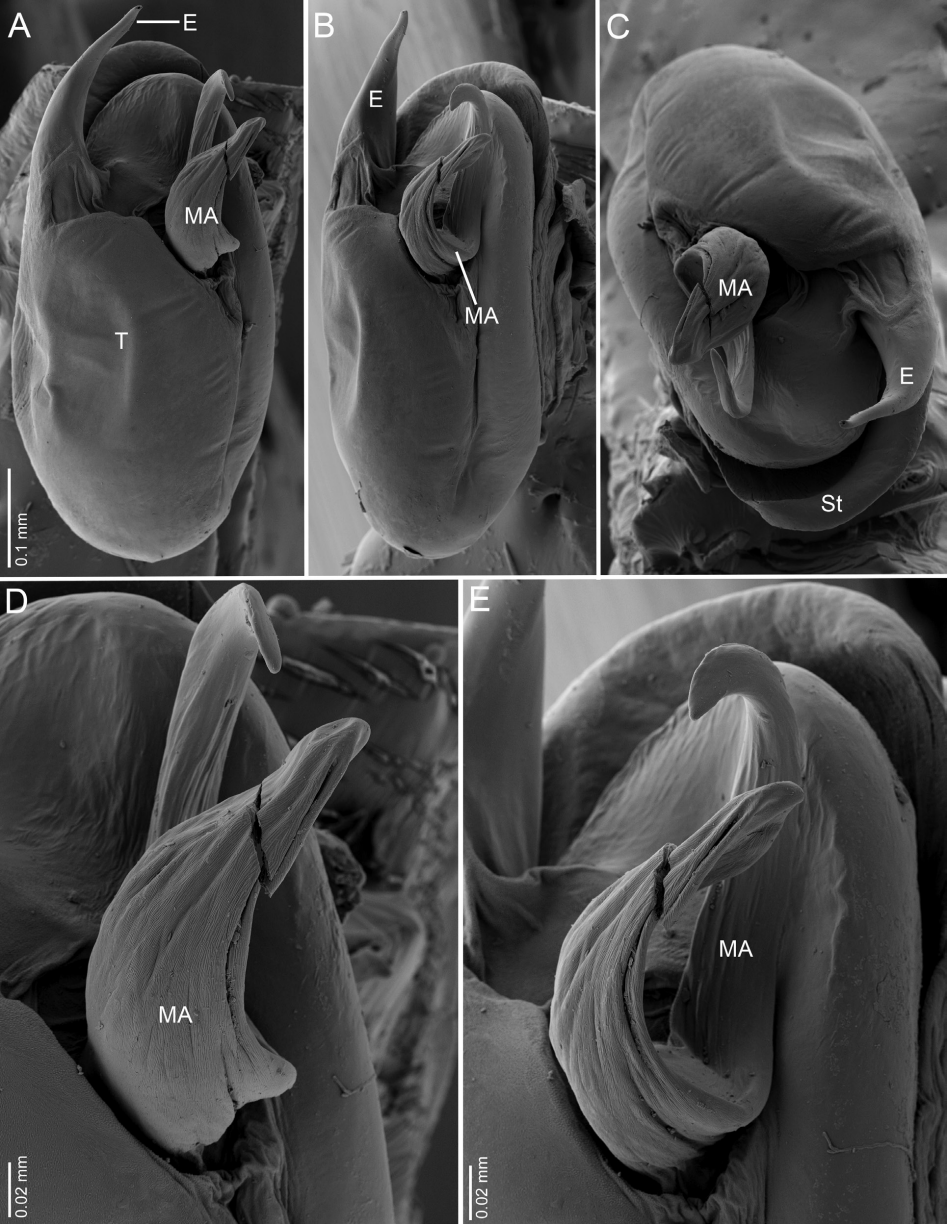
*Ilocomba marta* Brescovit, 1997, male (ICN-Ar 13819), left copulatory bulb, SEM images. A. Ventral view; B. Retrolateral view; C. Apico-ventral view; D. Median apophysis, ventral view; E. Median apophysis retrolateral view.

**Figure 8.**
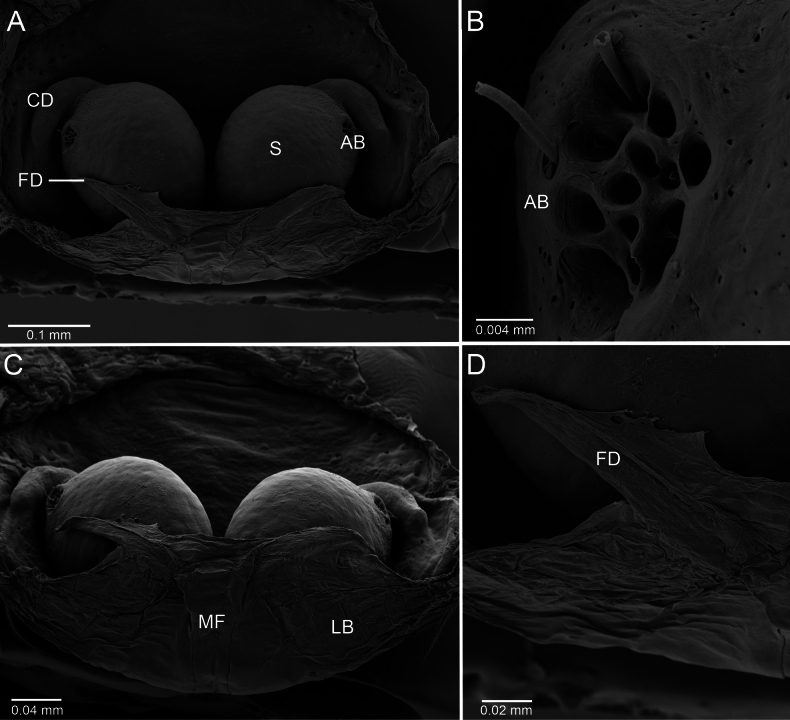
*Ilocomba marta* Brescovit, 1997, female (ICN-Ar 13818, genitalia, SEM images. A. Dorsal view; B. Accessory bulb; C. Posterior view; D. Fertilization duct.

Males of *Ilocomba marta* Brescovit, 1997 differ from those of *I. yotoco* Martínez and Cabra-García sp. nov. by the smaller, pointed retrolateral tibial apophysis (Fig. 6A–C) (vs larger, truncated at the tip in *I. yotoco*); the sub-squared cymbial projection, rugose and covered with granules (Figs 5B–E, 6A, 6B, 6D) (vs larger, curved, and horn-shaped in *I. yotoco*); and the ventral branch of the median apophysis, which is broad at the base, apically pointed, and bearing a small projection on the prolateral side of the base (Figs 5A, 7A–E) (vs laminar, wide, and strongly sclerotized in *I. yotoco*). Females differ from those of *I. yotoco* Martínez and Cabra-García sp. nov. and *I. perija* Brescovit, 1997 by the larger, spherical spermathecae (Figs 5F, 5G, 8A–D) (vs smaller and anteriorly elongated in *I. yotoco*); the thinner anterior copulatory ducts (vs wider and curved in *I. yotoco*); and the V-shaped sclerotization of the epigyne (vs sub-squared in *I. yotoco*).

**Figure 9.**
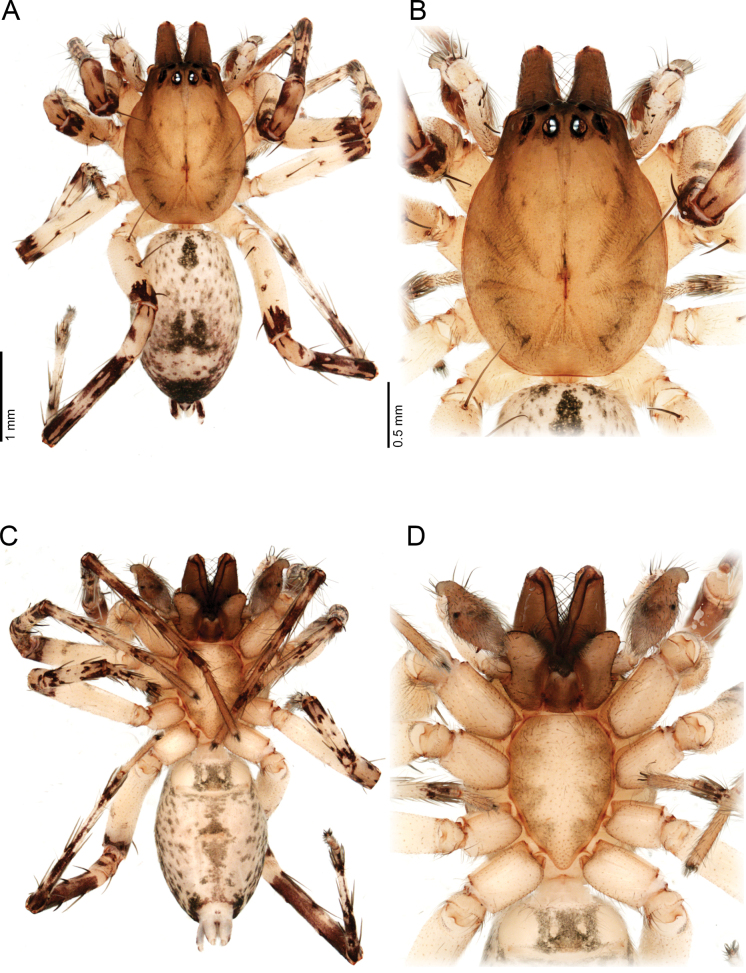
*Ilocomba marta* Brescovit, 1997, beta male (ICN-A 13819). A. Habitus, dorsal view; B. Carapace, dorsal view; C. Habitus, ventral view; D. Sternum.

##### Complementary description.

**Male** (ICN-Ar 13816). Coloration (Fig. 3A–D). Carapace brown, with two broad, lateral, dark longitudinal bands covered with black setae; margins bearing abundant white to yellowish setae, more conspicuous in live specimens. Cephalic region more pigmented, densely covered with white setae; black markings extending from posterior eyes toward cephalic region; eyes bordered with black. Fovea clothed with black setae. Chelicerae dark brown, with black basal markings. Endites, labium, and sternum brown; labium more pigmented; sternum bordered by a dark band, projecting triangularly toward center as paired dark triangles. Legs pale yellow; coxae slightly bordered with black; femora pale yellow at base, with numerous distal black spots on all segments; patellae to tarsi pale yellow-brown, with multiple dark spots, bands, and dots on all segments; legs overall densely setose. Abdomen pale beige, with a short, broad, median darkband on anterior dorsum; lateral regions with numerous dark dots extending to venter; medial region with two broad, longitudinal, dark markings; posterior region with a broad dark patch and lateral extensions. Venter beige; epigastric area with large dark patch; spiracle surrounded by dark pigmentation. Spinnerets pale beige, encircled by scattered dark patches. Palp. Retrolateral tibial apophysis very short, pointed (Fig. 6A–C). Cymbium almost as long as tibia; basal cymbial projection large, sub-squared, with finely rugose and granulate texture, composed of an irregular matrix of low reliefs and shallow depressions; slightly curved toward retrolateral side (Figs 5B–E, 6A–C). Tegulum longer than wide; sperm duct forming two loops (Figs 5A, 7A). Median apophysis with ventral branch very long, filiform, apically curved; dorsal branch strongly sclerotized, apically pointed, with a small projection at base of retrolateral edge (Figs 5A, 7A–E). Embolus long, with broad base, filiform, arising medially on tegulum (Figs 5A, 7A–C).

**Female** (ICN-Ar 13817). Coloration as in the male, but less pigmented (Fig. 4A–D). Epigynal plate sclerotized, with large, rounded copulatory openings situated anteriorly, and V-shaped lateral sclerotizations (Fig. 5F). Median field longer than wide, rectangular, delimited by broad and flat lateral lobes (Fig. 8C). Internally, copulatory ducts short, curved, and thin, positioned anteriorly (Fig. 5G). Spermathecae large, spherical, with prominent anterolateral accessory bulbs (Figs 5F, 8A, 8B). Fertilization ducts external, extending beyond the length of the spermathecae (Figs 5F, 8A, 8D).

##### Distribution.

Known from Sierra Nevada de Santa Marta, Magdalena and Cesar Departments, Colombia (Fig. 18).

##### Natural history.

Specimens of this species are abundant in low vegetation within cloud forest matrices in the Sierra Nevada de Santa Marta (Fig. 1A, B). Both sexes and juveniles are commonly collected together throughout the year. The species tends to be more abundant in secondary forests and in vegetation with dry leaves, where individuals construct retreats within the leaf layers.

#### Ilocomba yotoco

Taxon classificationAnimaliaAraneaeAnyphaenidae

Martínez & Cabra-García sp. nov.

56A17659-D68B-5B10-9654-39D210119298

https://zoobank.org/762EB147-FAA5-48A7-9EED-4EF16F73A225

**Figure 10.**
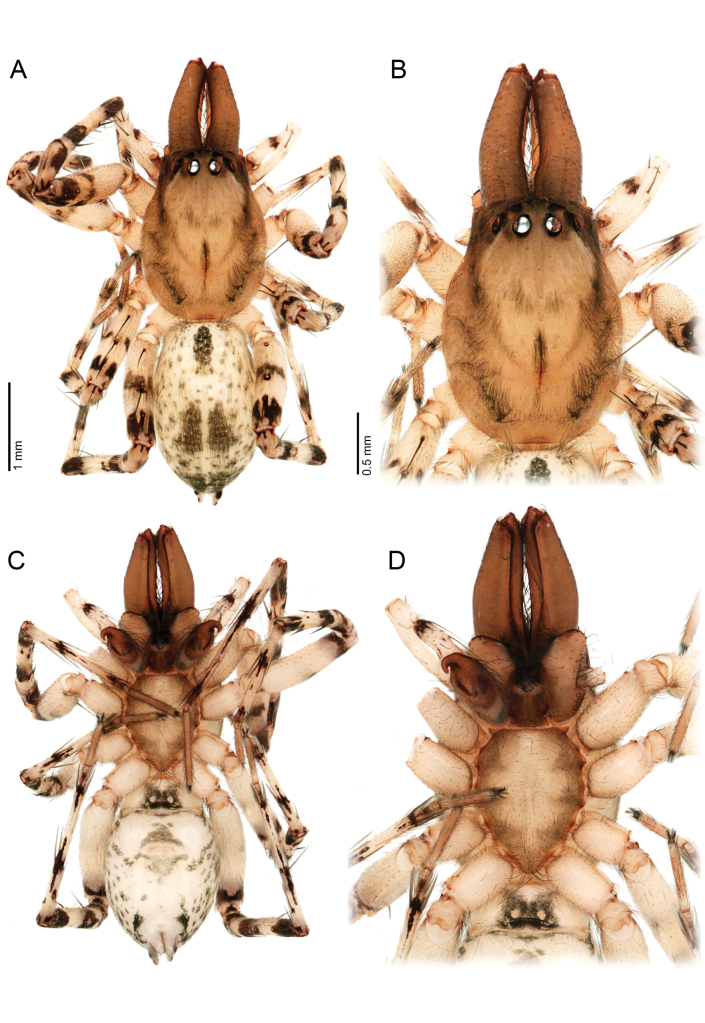
*Ilocomba yotoco* sp. nov., alpha male, holotype (MUSENUV-Ar 2256). A. Habitus, dorsal view; B. Carapace, dorsal view; C. Habitus, ventral view; D. Sternum.

**Figure 11.**
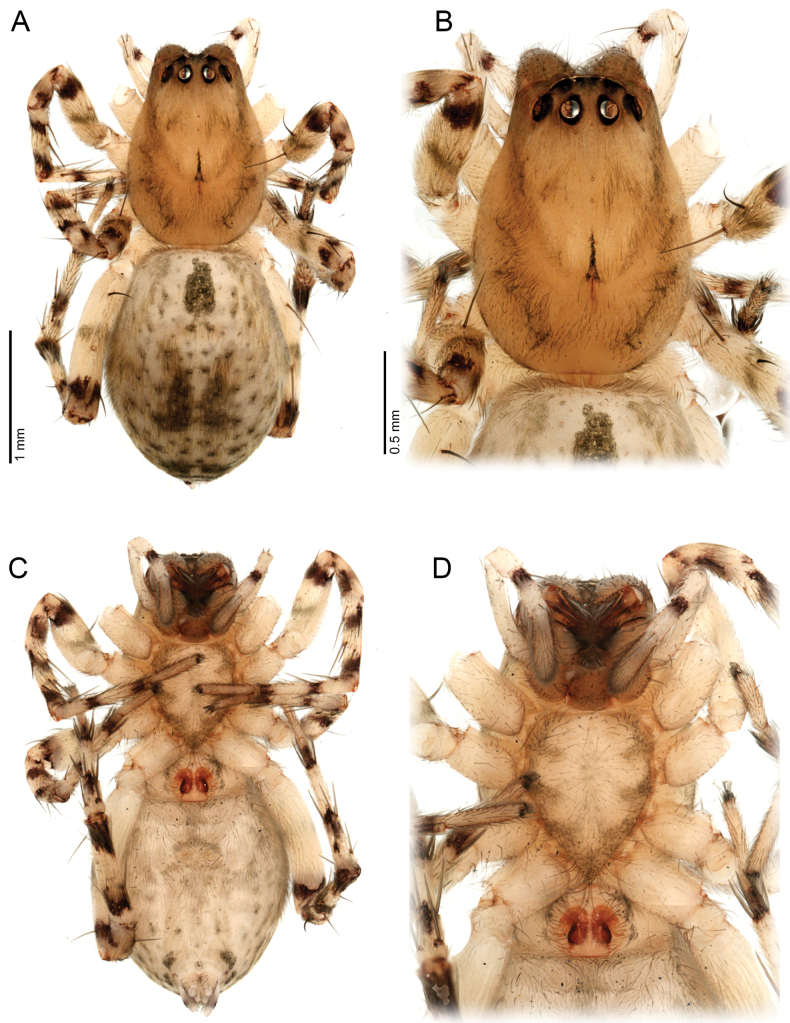
*Ilocomba yotoco* sp. nov., female, paratype (MUSENUV-Ar 2255). A. Habitus, dorsal view; B. Carapace, dorsal view; C. Habitus, ventral view; D. Sternum.

**Figure 12.**
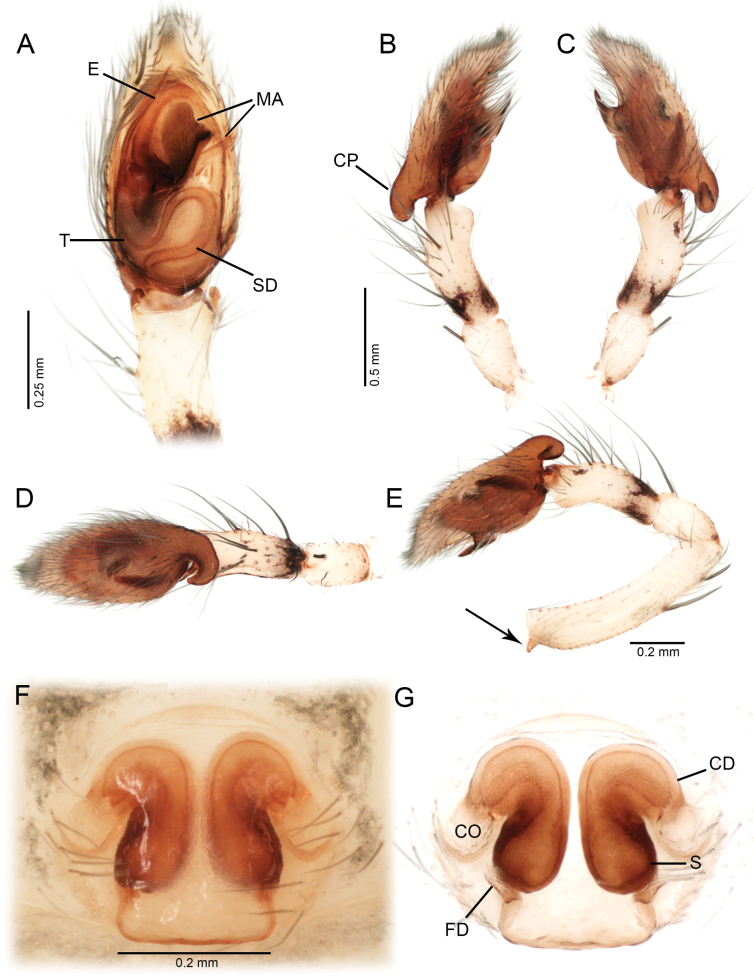
*Ilocomba yotoco* sp. nov., male, holotype (MUSENUV-Ar 2256), left palp. A. Ventral view; B. Prolateral view; C. Retrolateral view; D. Dorsal view; E. Retrolateral view, whole palp (arrow indicates a conical projection at dorsal end of tibia). Female (MUSENUV-Ar 2255), genitalia. F. Ventral view; G. Dorsal view.

**Figure 13.**
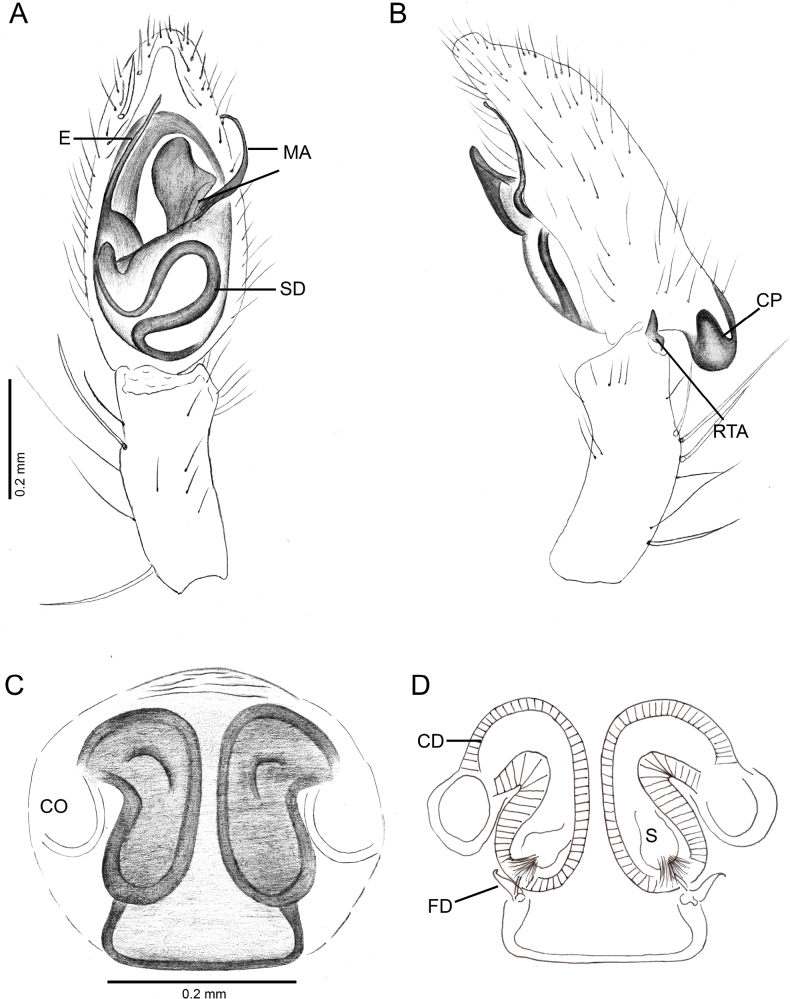
*Ilocomba yotoco* sp. nov., male, holotype (MUSENUV-Ar 2256), left palp. A. Ventral view; B. Retrolateral view. Female (MUSENUV-Ar 2255), genitalia. C. Ventral view; D. Dorsal view.

**Figure 14.**
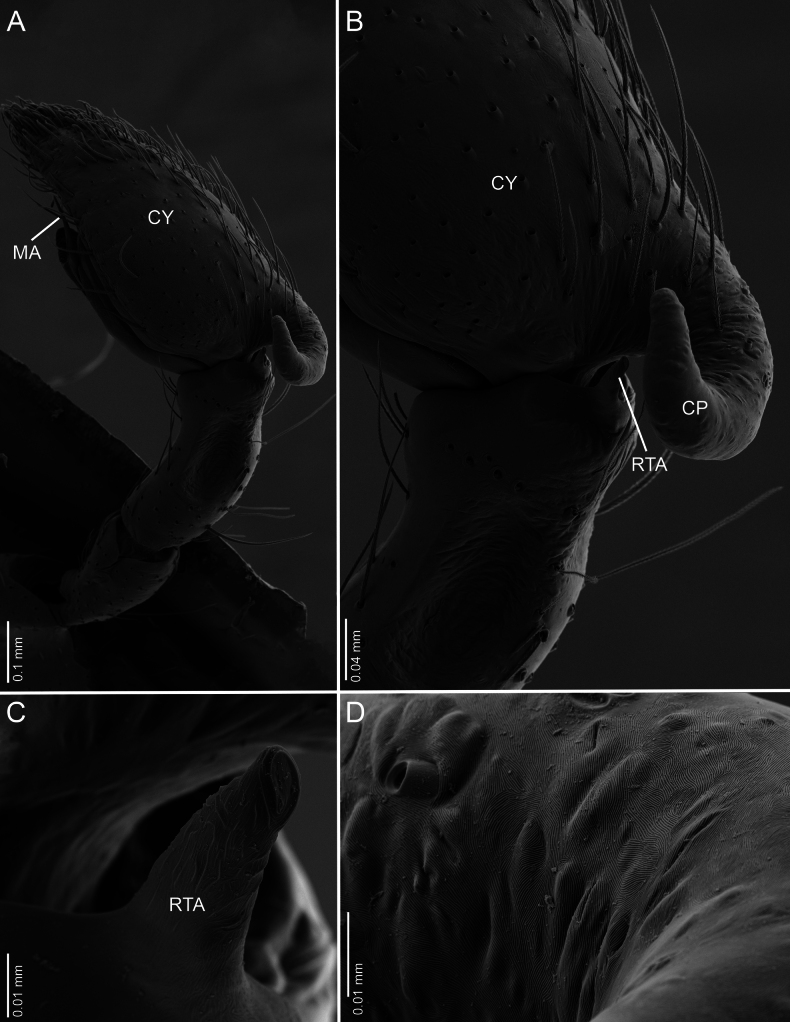
*Ilocomba yotoco* sp. nov., male (MUSENUV-Ar 2255; vchLAM-418), left palp, SEM images. A. Retrolateral view; B. Same, closed-up, details of the cymbial projection; C. Retrolateral tibial apophysis; D. Cuticular detail of cymbial projection.

**Figure 15.**
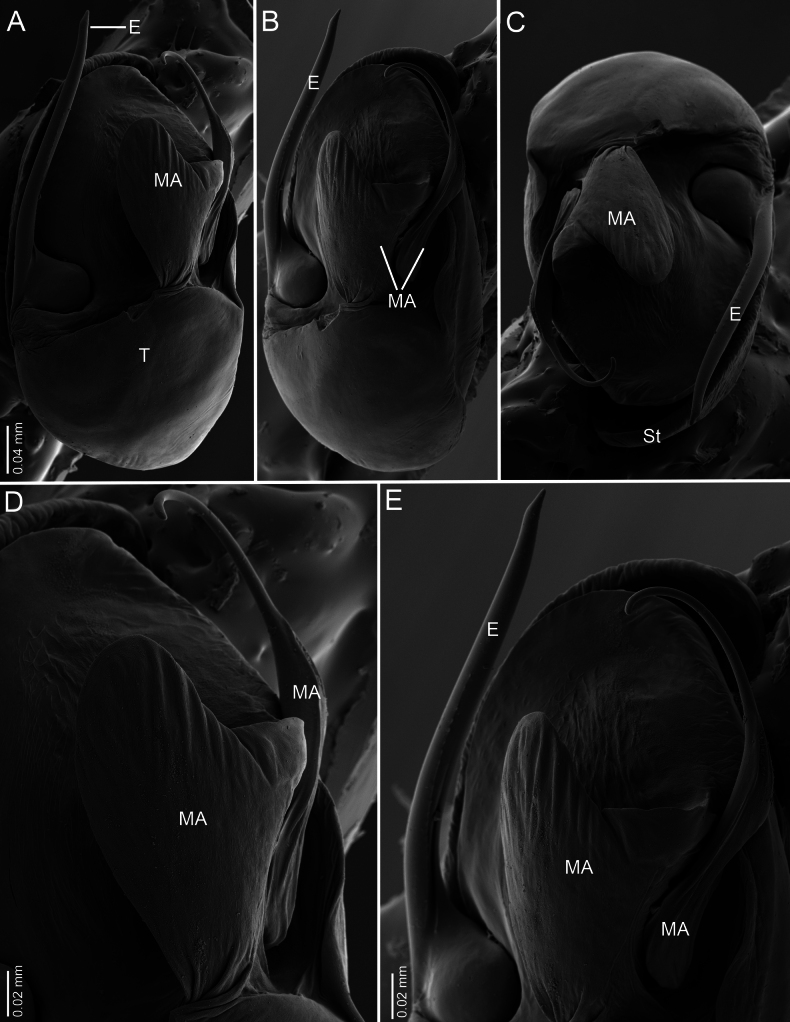
*Ilocomba yotoco* sp. nov., male (MUSENUV-Ar 2254), left copulatory bulb, SEM images. A. Ventral view; B. Retrolateral view; C. Apico-ventral view; D. Median apophysis, ventral view; E. Median apophysis retrolateral view.

Figs 10, 11, 12, 13, 14, 15, 17D–F

Ilocomba marta Brescovit, 1997: 83 (one male from Yotoco [3°50'N, 76°20'W], Valle del Cauca Colombia, XII.1976, W. Eberhad leg, deposited in MCZ, examined). Misidentification.

##### Type material.

***Holotype***. Colombia. 1 ♂ alpha; Valle del Cauca, Reserva Bosque Yotoco, sitio 2, parcela 1; (3°52'31.93"N, 76°26'9.36"W); alt. 1590 m.; 19 Sep. 2021; J. Cabra leg.; beating; MUSENUV-Ar 2256.

***Paratypes***. Same data as for holotype; beta male, 1 ♂ (MUSENUV-Ar 225); 1 ♀ (MUSENUV-Ar 2255); 1 ♂ (vchLAM-418, MUSENUV-Ar 2254); 1 ♀ (MUSENUV-Ar 2254).

##### Etymology.

The specific epithet yotoco refers to the Reserva Forestal Bosque de Yotoco, located in the department of Valle del Cauca, Colombia, where the type specimens were collected. The name also honors the reserve’s ongoing efforts to conserve the biodiversity in the Andean montane forest in Colombia. The name is used as a noun in apposition.

##### Diagnosis.

Males of *Ilocomba yotoco* Martínez and Cabra-García, sp. nov. can be distinguished from those of *I. marta* Brescovit, 1997 by the horn-shaped, curved basal process of the cymbium (Figs 12B–E, 13B, 14A, 14B) (vs wide, sub-squared, poorly projected, and bearing a small projection in *I. marta*); the larger retrolateral tibial apophysis with an acuminate tip (Fig. 14C) (vs poorly projected and apically pointed in *I. marta*); and the large, strongly sclerotized, scale-shaped ventral branch of the median apophysis (Figs 12A, 13A, 15A–E) (vs sharp, thin, and pointed in *I. marta*). Females can be distinguished from all other species in the genus by their anteriorly elongated spermathecae, broader copulatory ducts, and the squared sclerotized sculpture of posterior side of the epigyne (Figs 12F, 12G, 13C, 13D).

##### Description.

**Male** (holotype, MUSENUV-Ar 2256). Coloration (Fig. 10A–D). Carapace light brown, with two broad, lateral, longitudinal bands covered with black setae; margins bearing abundant white setae. Cephalic region more pigmented, densely covered with white setae; fovea clothed with black setae. Chelicerae dark brown. Endites, labium, and sternum brown; labium more pigmented; sternum bordered by a dark band, projecting triangularly toward center as paired dark triangles. Legs pale yellow; coxae slightly bordered with black. Femora pale yellow proximally, with dense dark mottling and scattered black patches distally on all legs. Patellae to tarsi pale yellow-brown, with multiple dark spots and incomplete transverse bands; markings more defined and abundant on legs I and II. Abdomen pale beige, with a short, broad, median dark band on anterior dorsum; medial region with two wide, longitudinal, dark bands; lateral regions bearing numerous dark dots, extending onto ventral surface; posterior region with a broad dark patch. Venter cream-colored; epigastric area with large dark patch; spiracle surrounded by dark pigmentation. Spinnerets pale beige, encircled by scattered dark patches. Total length 3.81, carapace length 1.91, width 1.37, height 0.90. Clypeus height 0.07. Eye diameters and interdistances: AME 0.06, ALE 0.11, PME 0.14, PLE 0.13; AME–AME 0.17, AME–ALE 0.22, PME–PME 0.38, PME–PLE 0.35, ALE–PLE 0.27. Chelicerae 1.11 long, four promarginal teeth, four retromarginal denticles, all restricted to the base. Sternum length 1.09, width 0.80. Leg measurements: leg I—femur 2.18/ patella 0.83/ tibia 2.44/ metatarsus 2.06/ tarsus 0.94/ total 8.45; II—2.09/ 0.85/ 2.44/ 2.08/ 0.94/ 8.40; III—1.31/ 0.47/ 0.90/ 1.25/ 0.37/ 4.30; IV—1.74/ 0.54/ 1.43/ 1.79/ 0.52/ 6.02. Leg spination: I—II tibia v 2-2-0, p 0-0-1, r 0-0-1, metatarsus d p1 m, v 2-0-0, p d1-d1-0, rd1-d1-0; III—IV tibia v 2-2-0, p 1-1, r 1-1, metatarsus d p1 m, v 2-0-2, p d1-1-2, r d1-1-2. Abdomen: length 2.07, epigastric furrow 0.44 from tracheal spiracle, spiracle 1.1 from base of spinnerets. Palp. Femur with conical projection dorsally (Fig. 12E, arrow). Retrolateral tibial apophysis short, pointed, dorsally displaced (Figs 12B–E, 13B, 14A, 14B). Cymbium almost as long as tibia; basal cymbial projection large, curved toward retrolateral side, with reticulations (Figs 12B–E, 13B, 14A, 14B). Tegulum longer than wide; sperm duct forming a single loop (Figs 12A, 13A, 15A–E). Median apophysis with dorsal branch very long, filiform, apically curved; ventral branch strongly sclerotized, laminar, distally expanded (Fig. 15A–E).). Embolus long, with broad base, filiform, arising medially on tegulum (Figs 12A, 13A, 15A–E).

**Female** (Paratype, MUSENUV-Ar 2254). Coloration as in the male, but less pigmented (Fig. 11A–D). Total length 3.42, carapace length 1.46, width 1.11, high 0.71. Clypeus height 0.03. Eye diameters and interdistances: AME 0.05, ALE 0.10, PME 0.11, PLE 0.09; AME–AME 0.13, AME–ALE 0.17, PME–PME 0.29, PME–PLE 0.28, ALE–PLE 0.20. Chelicerae 0.54 long, four promarginal teeth, four retromarginal denticles. Sternum length 0.87, width 0.64. Leg measurements: leg I—femur 1.31/ patella 0.58/ tibia 1.34/ metatarsus 1.11/ tarsus 0.55/ total 4.89; II—0.89/ 0.49 / 0.85/ 0.79 / 0.43/ 3.45; III—0.84/ 0.40/ 0.65/ 0.75/ 0.35/ 2.99; IV—1.41/ 0.49/ 1.13/ 1.25 / 0.41/ 4.69. Leg spination: I—II tibia v 2-2-0, p 1-1-0, r 1-1-0, metatarsus d p1, v 2-0-0, p d1-d1-0, rd1-d1-0; III—tibia d r 1-0-0, v 2-0-2, p 0-1-1, r 0-1-1, metatarsus d p1 m, v 2-0-2, p 0-1-2, r 0-1-2; IV—tibia d 1-0-0, v r 1-r1-2, p 0-1-1, r 0-1-1, metatarsus d p1 m, 2-0-2, p 1-1-2, r 1-1-2. Abdomen: length 2.03, epigastric furrow 0.40 from tracheal spiracle, spiracle 1.25 from base of spinnerets. Epigynal plate sclerotized, with large, rounded copulatory openings situated anteriorly (Fig. 12F). Posterior region squared, with sclerotized margins (Fig. 12F). Internally, copulatory ducts short, curved, very broad, positioned anteriorly (Figs 12G, 13D). Spermathecae large, elongate anteriorly. Fertilization ducts shorter than spermathecae (Figs 12G, 13D).

##### Variation.

Males (*n* = 4): total length: 3.05–3.81; carapace length: 1.51–1.98. Females (*n* = 2): total length: 3.11–3.42; carapace length: 1.35–1.46. Brown tones may appear darker in some specimens; the pattern of markings may vary slightly in distribution, and the spots sometimes appear black rather than dark green. No variation in genital morphology was observed between alpha and beta males.

##### Distribution.

Only known from Valle del Cauca department, Colombia (Fig. 18).

**Figure 16.**
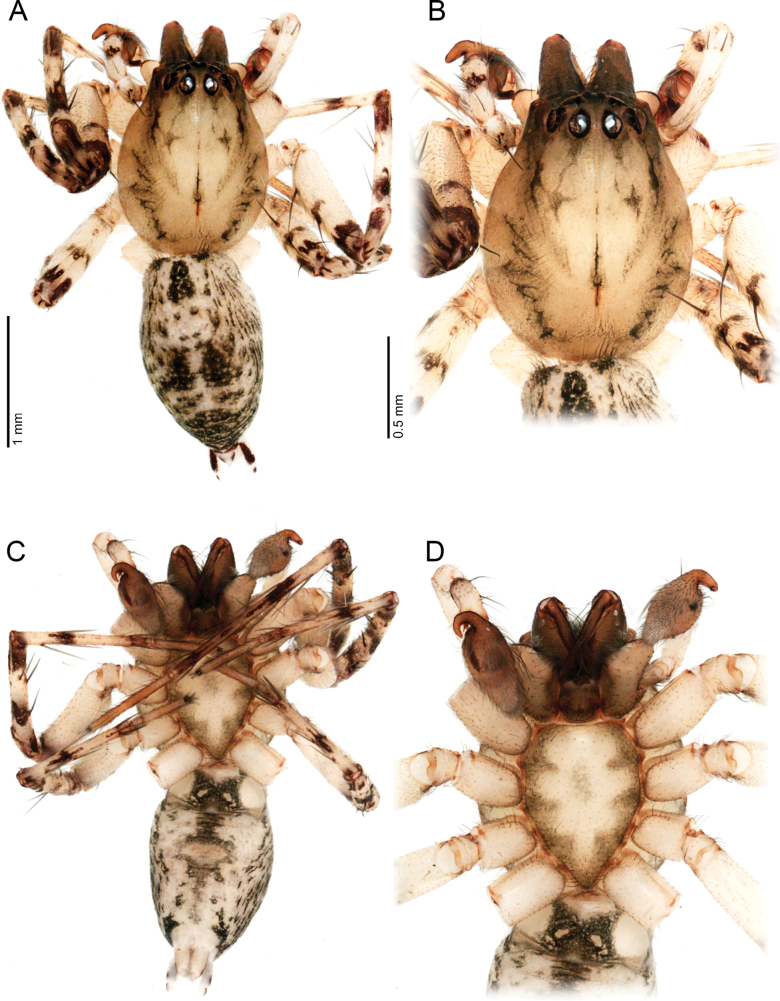
*Ilocomba yotoco* sp. nov., beta male (MUSENUV-Ar 2254). A. Habitus, dorsal view; B. Carapace, dorsal view; C. Habitus, ventral view; D. Sternum.

**Figure 17.**
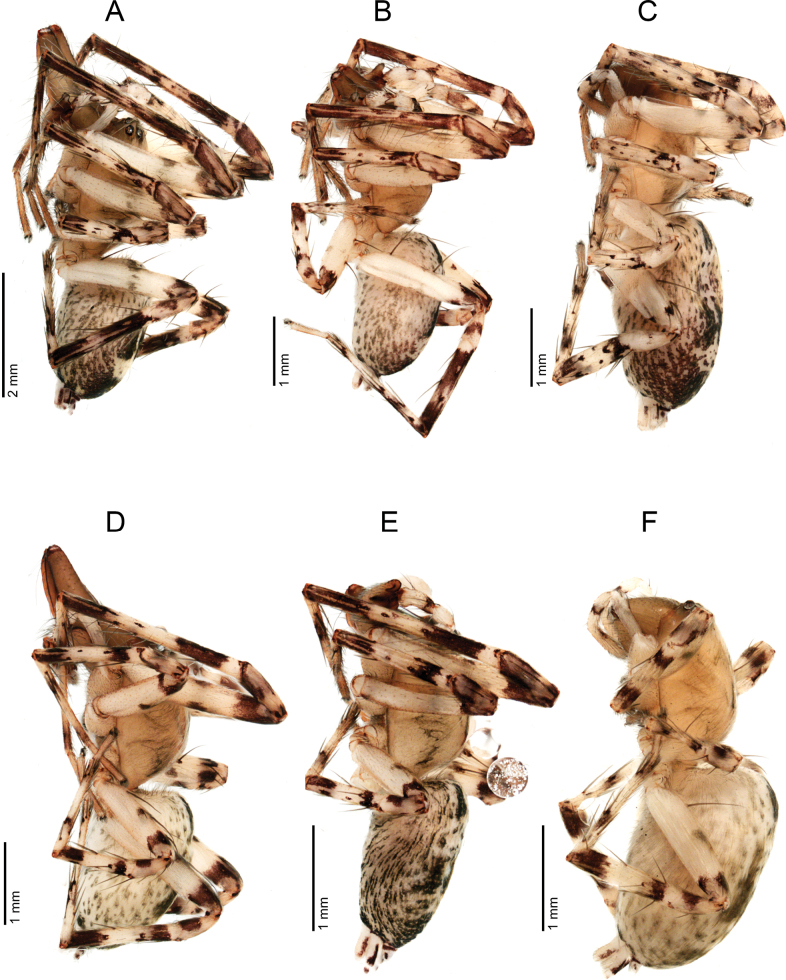
*Ilocomba marta* Brescovit, 1997, lateral view. A. Alpha male (ICN-Ar 13816; vchLAM-00308); B. Beta male (ICN-Ar 13819); C. Female (ICN-Ar 13817; vchLAM-00309). *Ilocomba yotoco* sp. nov., lateral view. D. Alpha male, holotype (MUSENUV-Ar 2256); E. Beta male, paratype (MUSENUV-Ar 2254); F. Female, paratype (MUSENUV-Ar 2255).

**Figure 18.**
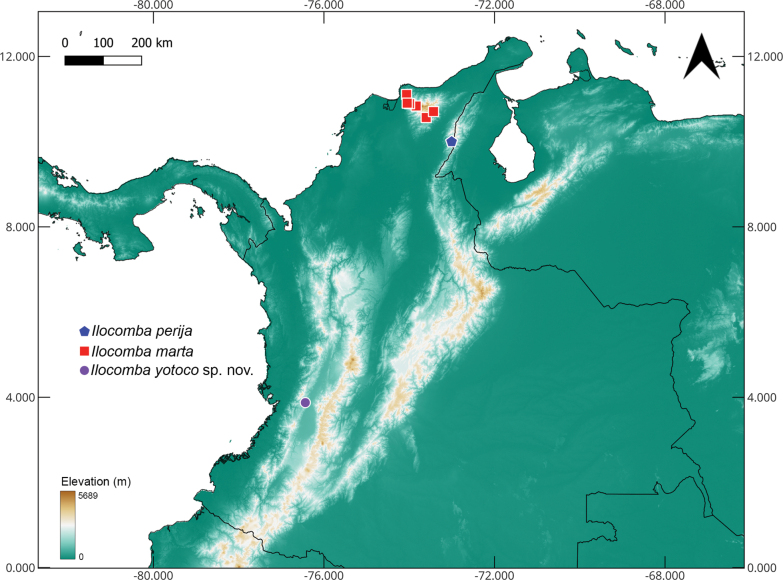
Distribution map of *Ilocomba* species in Colombia. Background shows elevation gradient in meters.

## Discussion

Both maximum-likelihood and parsimony analyses recovered *Ilocomba* as a monophyletic genus, with *I. yotoco* sp. nov. consistently placed as the sister to *I. marta* (Fig. 2). Our ML analysis suggests *Hatitia* as sister to *Ilocomba*, in contrast to Oliveira’s (2023) morphological study, which recovered a sister relationship with *Jessica* under implied weights (*k* = 7) and with *Katissa* under equal weights. Although our analysis is not fully comparable to Oliveira’s (2023), as we included only 10 Anyphaeninae genera with available molecular data, the combined evidence from both studies suggests that the sister group of *Ilocomba* remains unresolved and that further studies incorporating additional molecular data will be necessary to clarify this issue.

The discovery of *I. yotoco* sp. nov. underscores the value of integrating molecular data with traditional morphological approaches in the study of Colombia’s underexplored montane forests. The new species consistently differs from *I. marta* and *I. perija* in genitalic morphology, and these differences, together with its disjunct geographic distribution and interspecific COI distances, provide robust support for species delimitation under an integrative taxonomic framework.

Our examination of multiple individuals of *I. yotoco* sp. nov. and *I. marta* revealed two discrete male morphotypes—alpha and beta—distinguished primarily by cheliceral length and anterior leg setation, while sharing highly similar copulatory structures. This pronounced intrasexual dimorphism has been observed in other Anyphaeninae such as *Italaman* Brescovit, 1997 and *Tafana* Simon, 1903 (Brescovit 1997; Oliveira and Brescovit 2021), and may reflect alternative reproductive strategies, such as those described in other Araneae lineages (Foellmer and Fairbairn 2005; Funke and Huber 2005; McLean et. al. 2018). Alpha males, characterized by enlarged chelicerae and longer legs, may be involved in male–male combat or mate guarding, whereas beta males—morphologically more similar to females—might adopt sneaker strategies (Solano-Brenes et al. 2018; Abhijith et al. 2022). Behavioural and experimental studies will be necessary to test the functional significance and evolutionary stability of these dimorphic traits.

The description of *I. yotoco* sp. nov. substantially expands the known distribution of the genus, now including a locality in the western Andes of Colombia. This geographically disjunct distribution, relative to *I. marta* in the Sierra Nevada de Santa Marta and *I. perija* in the Serranía de Perija, suggests that additional *Ilocomba* lineages may occur in the intervening Andean cordilleras and adjacent regions. Continued sampling efforts in poorly surveyed montane habitats are likely to yield further taxonomic novelties.

By integrating molecular and morphological evidence this study provides a refined systematic framework for *Ilocomba* and underscores the importance of Colombia’s mountainous ecosystems as reservoirs of undiscovered spider diversity.

## Supplementary Material

XML Treatment for Ilocomba

XML Treatment for Ilocomba marta

XML Treatment for Ilocomba yotoco

## Additional information

### Use of AI

No use of AI was reported.

### Author ORCIDs

Leonel Martínez https://orcid.org/0000-0002-4166-0561

Melisa Eyes-Escalante https://orcid.org/0000-0002-8707-0743

Jimmy Cabra-García https://orcid.org/0000-0001-9263-4390

### Data availability

All of the data that support the findings of this study are available in the main text or Supplementary Information.

## Supplementary materials

Supplementary material 1 Molecular datasets and alignment files for phylogenetic analyses of *Ilocomba* sp. nov. Data type zip Explanation note This supplementary material includes sequence data in FASTA and GenBank formats for the mitochondrial (COI, 16S) and nuclear (28S, H3) genes used in the phylogenetic analyses. It also contains a Python script (phyloconvert.py) used for dataset formatting and conversion. https://binary.pensoft.net/file/1602776This dataset is made available under the Open Database License (http://opendatacommons.org/licenses/odbl/1.0/). The Open Database License (ODbL) is a license agreement intended to allow users to freely share, modify, and use this Dataset while maintaining this same freedom for others, provided that the original source and author(s) are credited. Leonel Martínez and Jimmy Cabra-García

Supplementary material 2 Aligned sequences and concatenated dataset used in phylogenetic analyses of *Ilocomba* yotoco sp. nov. Data type zip Explanation note This supplementary material includes aligned nucleotide sequences for the mitochondrial (COI, 16S) and nuclear (28S, H3) markers used in the phylogenetic analyses of *Ilocomba*. Files are provided in FASTA format, including:Raw alignments (*_aln.fasta) Trimmed alignments (*_aln_pru_mod.fasta) and a concatenated matrix (concatenated.fas) used in the combined analyses. https://binary.pensoft.net/file/1602777This dataset is made available under the Open Database License (http://opendatacommons.org/licenses/odbl/1.0/). The Open Database License (ODbL) is a license agreement intended to allow users to freely share, modify, and use this Dataset while maintaining this same freedom for others, provided that the original source and author(s) are credited. Leonel Martínez and Jimmy Cabra-García

Supplementary material 3 Phylogenetic trees and analysis files from parsimony and likelihood inference of *Ilocomba yotoco* sp. nov. Data type zip Explanation note This supplementary material includes the output files, tree searches, and scripts used for the phylogenetic analyses conducted using parsimony (TNT) and maximum likelihood (IQ-TREE). It contains: 1) Folders and files from the parsimony analyses generated in TNT (concat.tnt, concat.run, .tre, .txt, and .nwk tree files). 2) Folders containing the maximum likelihood replicates and best trees. 3) Final tree files in Newick (.nwk) and graphical (.pdf) formats. https://binary.pensoft.net/file/1602778This dataset is made available under the Open Database License (http://opendatacommons.org/licenses/odbl/1.0/). The Open Database License (ODbL) is a license agreement intended to allow users to freely share, modify, and use this Dataset while maintaining this same freedom for others, provided that the original source and author(s) are credited. Leonel Martínez and Jimmy Cabra-García
