# Supplementary figures and images for: Morphological and molecular evidence support a new species of the genus Ilocomba Brescovit, 1997 (Araneae, Anyphaenidae, Anyphaeninae) from the Andes of Colombia
Source: Zookeys. 2026 Apr 22;1278:19–51. doi: 10.3897/zookeys.1278.162601 (PMC13129551; doi:10.3897/zookeys.1278.162601)

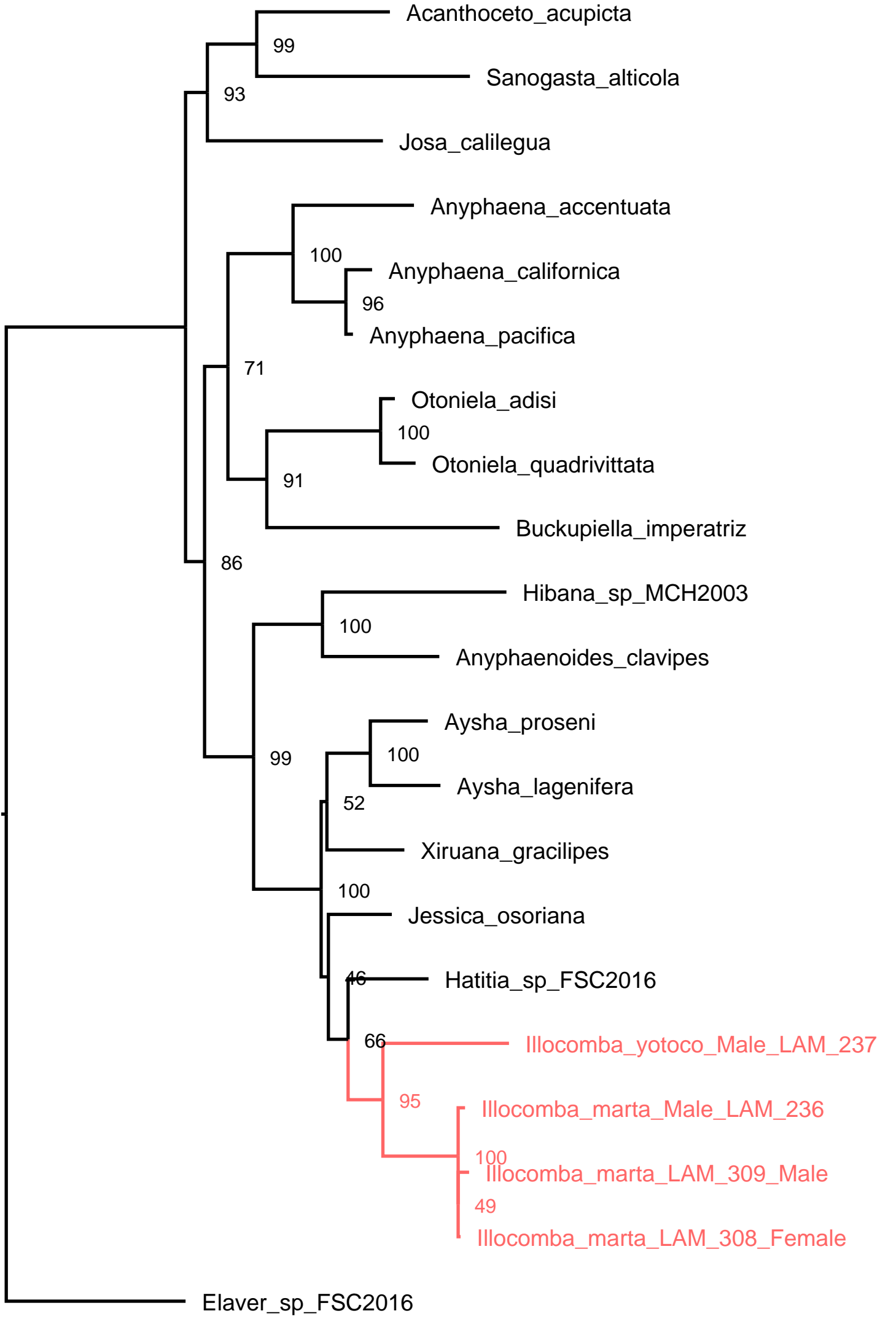

0.06

Supplement: Supplementary material 3 — Phylogenetic trees and analysis files from parsimony and likelihood inference of Ilocomba yotoco sp. nov. [file zookeys-1278-019_article-162601__-s003.zip › 2_Phylo/ML/10_-11581.573762_Best/ml.pdf]

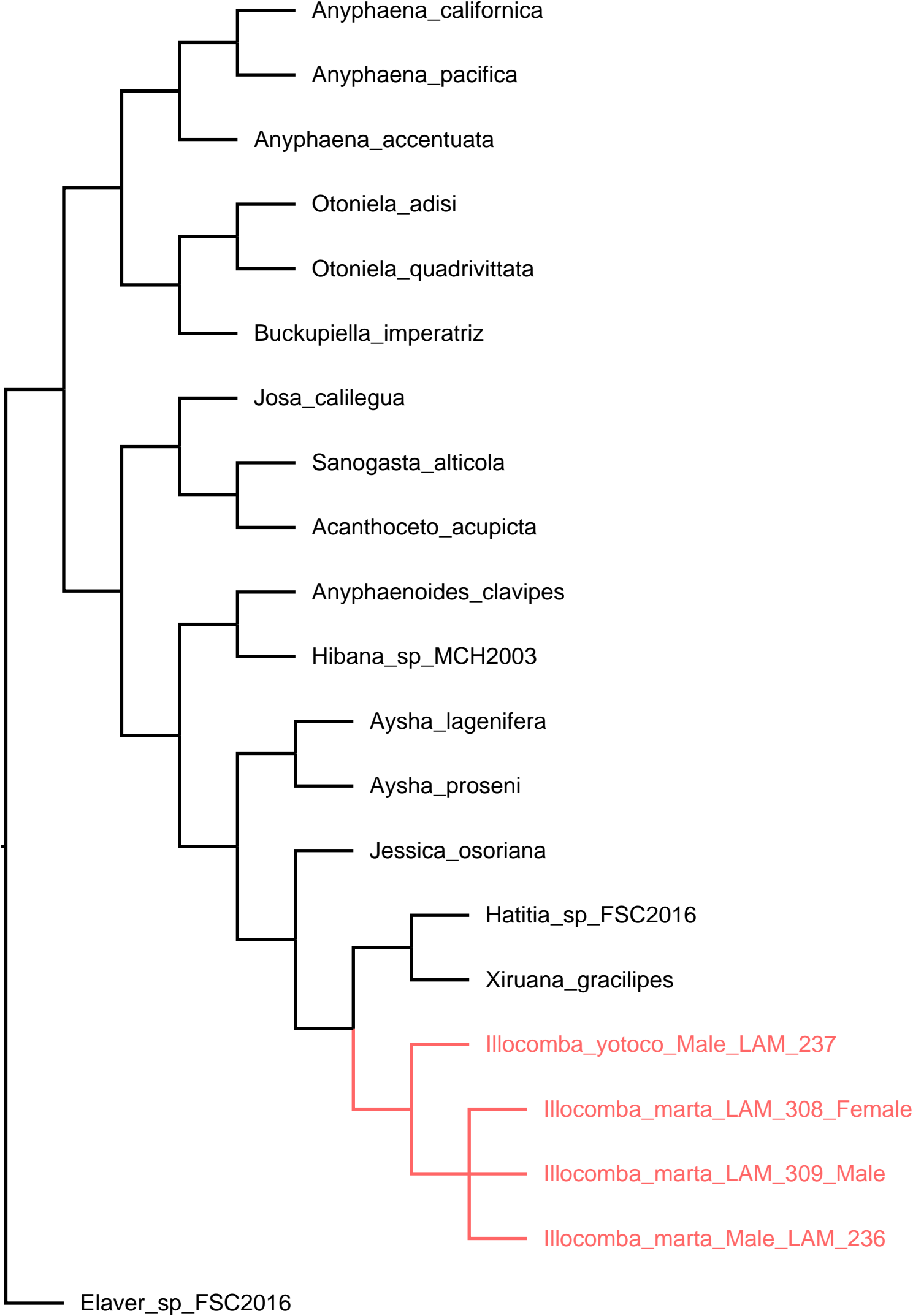

1.0

Supplement: Supplementary material 3 — Phylogenetic trees and analysis files from parsimony and likelihood inference of Ilocomba yotoco sp. nov. [file zookeys-1278-019_article-162601__-s003.zip › 2_Phylo/MP/tnt_ew.nwk.pdf]
